# Factors That Cause Concerns after Cardioverter Defibrillator Implantation

**DOI:** 10.3390/ijerph18116095

**Published:** 2021-06-05

**Authors:** Olimpia Karczewska, Agnieszka Młynarska

**Affiliations:** 1 Department of Anaesthesia and Intensive Nursing Care, Faculty of Health Sciences in Katowice, Medical University of Silesia, 40-752 Katowice, Poland; 2 Department of Gerontology and Geriatric Nursing, Faculty of Health Sciences in Katowice, Medical University of Silesia, 40-635 Katowice, Poland; amlynarska@sum.edu.pl

**Keywords:** implantable cardioverter defibrillator, concerns, anxiety

## Abstract

Background and Objectives: The aim of the study was to assess the factors that influence the occurrence of concerns and their intensification after the implantation of a cardioverter defibrillator. Materials and Methods: This was a prospective and observational study including 158 patients. The study was conducted in two stages: stage I before implantable cardioverter defibrillator (ICD) implantation and stage II follow-up visit six months after ICD implantation. Standardized questionnaires were used in both stages. Results: Age and female gender were significantly correlated with the occurrence and intensity of concerns. Patients who had a device implanted for secondary prevention also experienced higher levels of concern. Additionally, a multiple regression model using the stepwise input method was performed. The model was statistically significant and explained 42% of the observed variance in the dependent variable (*p* = 0.0001, R^2^ = 0.4215). The analysis showed that age (*p* = 0.0036), insomnia (*p* = 0.0276), anxiety (*p* = 0.0000) and negative emotions (*p* = 0.0374) were important predictors of the dependent variable and enabled higher levels of the number of concerns to be predicted. Conclusions: There is a relationship between the severity of the concerns related to an implanted ICD and age, gender, anxiety, negative emotions and insomnia. Indications for ICD implantation may be associated with increased concerns about ICD.

## 1. Introduction

Sudden cardiac death (SCD) is defined as a death from cardiac causes that occurs within the first hour after the onset of symptoms. Since pharmacological treatment is not always effective and surgery can only be performed on some patients, cardioverter defibrillator (ICD) implantation has become the treatment of choice for people who are at risk of SCD [1,2]. Mieczysław Mirowski was the creator of the idea of a treatment with a device that can record the heart rhythm and react in the event of ventricular tachycardia or fibrillation. In an animal laboratory, he implanted a defibrillator in a dog. The experiment to induce ventricular fibrillation in the animal, which led to a discharge from the device, was recorded with a camera. The report and the film were published, but they were not approved by the scientific community. The first successful human implantation was performed in 1980. Since then, the implantable cardioverter defibrillator has become world famous. Over time, the device has been expanded, and its diagnostic and therapeutic possibilities have been improved [3,4].

The criterion for the qualification for ICD implantation is the fulfillment of the conditions specified in the ESC (European Society of Cardiology) guidelines. The indications for ICD implantation are divided into indications for secondary prevention—in patients after cardiac arrest as a result of ventricular fibrillation or sustained ventricular tachycardia—and indications for primary prevention—in patients without documented malignant arrhythmias but who are in the high-risk group for SCD. The issue of primary prevention is more complex and concerns a larger number of patients, including those with coronary artery disease, nonischemic dilated cardiomyopathy, advanced heart failure, hypertrophic cardiomyopathy and arrhythmogenic right ventricular cardiomyopathy and patients with long QT syndrome, Brugada syndrome or polymorphic ventricular tachycardia [1,5]. Although the benefits of ICD therapy are unequivocal, the treatment with this method is associated with some psychosocial consequences. It is often associated with the occurrence of anxiety and depression; in some patients, stress may take a more intense form, such as post-traumatic stress disorder (PTSD).

Patients with a high level of anxiety have a greater tendency to experience such problems. Since one condition of good treatment is a compromise between achieving the therapeutic goal and the impact of therapy on the quality of life, it is necessary to pay special attention to patients after ICD implantation. The early identification of high-risk patients in terms of disorders such as anxiety or depression would enable the appropriate cardiac rehabilitation to be implemented and integrate them with psychological support, which would reduce anxiety and improve their quality of life [6]. Moreover, patients who are aware of how an ICD works tolerate the pain and discomfort better and also experience less anxiety related to the discharge [7].

The aim of this study was to assess the factors that influence the occurrence of concerns and their intensification after the implantation of an implantable cardioverter defibrillator.

## 2. Materials and Methods

### 2.1. Study Design and Setting

This was a prospective and observational study including 158 patients (31 women and 127 men, average age 67.6 ± 9.1) who were admitted for ICD insertion between November 2018 and February 2020.

The minimum sample size was 147, which was calculated based on the available patient population with a 95% confidence interval. The data to determine the minimum number of individuals within the group was obtained from the EHRA White Book 2017 [8].

### 2.2. Study Participants and Selection

The inclusion criteria for the study followed the 2015 ESC guidelines for the management of patients with ventricular arrhythmia and the prevention of sudden cardiac death. The exclusion criteria were a previously diagnosed mental illness, cancer in the active phase, incomplete data, advanced heart failure suitable for resynchronization therapy, a previously implanted antiarrhythmic device and failure to attend a follow-up appointment.

This study was conducted in two stages.

- Stage I aimed at a clinical examination with data collection and analysis before ICD implantation. Additionally, specific questionnaires were completed (DS-14 scale—Scale for Measuring a Type-D Personality, Acceptance of Illness Scale (AIS), Hospital Anxiety Depression Scale (HADS) and the Athens Insomnia Scale (AIS)).
- Stage II consisted of a follow-up appointment after six months ± two weeks following ICD insertion. The occurrence of any ICD interventions, hospital admissions or complications were reviewed. The previous questionnaires were again completed, including the ICD Patient Concern Questionnaire (ICDC) [9].

The current study was a subgroup analysis of a larger project focused on factors related to ICD concerns.

### 2.3. Ethical Considerations

The study protocol was compliant with the Helsinki Declaration, and it was approved by the Bioethics Committee of Silesian Medical University in Katowice (Resolution KNW/0022/KB/224/I/18). Participation in the study was anonymous and voluntary. Before enrolment, participants were informed about study confidentiality, its purpose and methodology. They were also given the choice to withdraw from the study if they wished to do so. Finally, informed consent was obtained.

### 2.4. Research Instruments

The personality type was assessed using the DS-14 scale, the acceptance of the disease, as well as the occurrence of symptoms of anxiety and depression, insomnia and the severity of concerns related to the implanted ICD, were assessed in all of the patients who were included in the study.

The ICD Patient Concerns Questionnaire (ICDC) is a standardized tool that can be used to assess the concerns of patients with an implanted ICD. It contains 20 questions, which are scored on a 5-point Likert scale: from 0—not applicable to 4 points, which indicates a high intensity. The total score on the scale ranges from 0 to 80 points. The greater the number of points, the greater the severity of fears, and the more serious the fears about living with an ICD are. The number and severity of concerns can be combined to obtain a total score up to 100. The scale also has two subscales—factor 1 assesses the perceived limitations, and factor 2 assesses device-specific concerns. The internal consistency, which was measured as Cronbach’s alpha, showed the optimal results for the whole questionnaire (0.96) [9,10,11].

The Scale for Measuring a Type-D Personality, the DS-14 scale, is used to measure the severity of the type D stress personality traits in adults. It consists of 14 statements: 7 refer to the tendency to experience negative emotions (negative emotionality), while the other 7 refer to refraining from expressing these emotions and related behaviors (social inhibition). Individual statements were assessed using a 5-point scale: 0—false, 1—rather false, 2—hard to say, 3—rather true and 4—true. A score greater than or equal to 10 points for each dimension indicates the presence of a type D personality. A score greater than or equal to 10 points in one of the dimensions indicates an intermediate personality type. A score lower than 10 points in both dimensions means the patient does not have a type D personality. The scale is highly reliable—the Cronbach’s alpha coefficient was 0.88 for the negative emotion subscale, whilst it was 0.84 for social inhibition [12].

The Acceptance of Illness scale (AIS) is a tool designed to measure the degree of disease acceptance in adults. It can be used for any disease. It contains eight statements that describe the negative consequences of poor health, such as any limitations imposed by the disease, lack of self-sufficiency, a sense of dependence on third parties and lowered self-esteem. Strong agreement with a given statement (grade 1) indicates a bad adaptation to the disease, whereas strong disagreement (grade 5) means acceptance of the disease. The higher the point value, the better the acceptance; a low value indicates a lack of acceptance and a strong sense of mental discomfort. Eight to 18 points indicates a lack of acceptance of the disease, 19–29 points indicates an average level of acceptance of the disease and 30–40 points indicates an acceptance of the health situation at a good level. The Polish version of the AIS has a high Cronbach’s alpha coefficient—0.82. It was also in accordance with the original version of the scale [13].

The Hospital Anxiety Depression Scale (HADS) is a tool for the assessment of anxiety and depression with a focus on negative emotions. The omission of items related to somatic complaints has made the HADS scale one of the most commonly used screening tests in medical settings. The scale measures a condition, not a feature. It is composed of seven statements related to anxiety and seven statements related to depressive states. Each of the statements can have a score from 1 to 3 points. The lower the score, the lower the severity of the disorder. 0–7 points indicates no disorders, 8–10 points—a borderline state and 11–21 points—mood disorders. The scale is characterized by both a high sensitivity and specificity [14]. The internal consistency, which is measured as a Cronbach’s alpha, indicates optimal results for both the subscales and for the whole questionnaire (0.88) [15].

The Athens Insomnia Scale (AIS) is a self-reporting tool comprising eight statements about the various symptoms of insomnia. Each item is assessed on a scale of 0–3 points, where 0 means no symptom and 3—a significant severity. The total score on the scale is in the range of 0–24 points; more points are considered the value that enables the occurrence of insomnia to be concluded with a high probability. The AIS is one of the most commonly used scales, both for diagnostic purposes and in research into the effectiveness of treating insomnia. The original validation studies demonstrated the high reliability and validity of this tool [16].

### 2.5. Statistical Analyses

The quantitative variables were analyzed by calculating the mean, standard deviation, median, quartiles, minimum and maximum. The analysis of the qualitative variables was analyzed by calculating the number and percentage of the occurrences of each value. The analysis of the survey questions was performed by calculating the number and percentage of the occurrences of each answer. The values of the quantitative variables in the two groups were compared using the Mann–Whitney test. A significance level of 0.05 was adopted in the analysis. Thus, all the *p*-values below 0.05 were interpreted as showing significant dependencies. The Spearman correlation coefficient r was used to correlate a concern about ICD implantation and insomnia, the symptoms of anxiety and depression and the number of discharges. In order to assess whether the analyzed parameters were predictors of the dependent variables, a multiple regression analysis using the stepwise method was used. The analysis was performed using R software, version 4.0 (R core Team, Vienna, Austria) [17].

## 3. Results

All patients were reviewed at follow-up. Most patients (82.91%) lived in large urban areas, 70.2% were in a relationship, 90% had children and 83.5% lived with their immediate families (spouse or partner, children or grandchildren). The vast majority denied cigarette smoking (76.5%) and alcohol consumption (97.5%). ICD insertion was indicated for primary prevention in 85% of the patients. The detailed characteristics of the study group are presented in Table 1.

The average number of concerns in all the examined patients was 7.56 ± 4.96. Exact data about specific concerns are presented in Table 2. 

The analysis of the impact of age on the severity of anxiety showed a significant correlation with all the subscales of the ICDC questionnaire. The older the patient, the lower the level of anxiety after ICD implantation. The details are presented in Table 3.

Comparison of the number of fears, their severity and other components of the ICDC scale showed an intensity of the effect, which was statistically more significant in women than in men. The details are presented in Table 4.

Place of residence, marital status, offspring, level of education, stimulants, BMI, EF, NYHA, SBP, DBP and HR indicated that they had no statistically significant influences on the number and severity of concerns after ICD implantation.

The indication for ICD implantation was a significant factor that influenced the severity of a patients’ anxiety. Patients who had an ICD implanted for secondary prevention had a higher level of anxiety. No differences were found that were dependent on prevention of the implanted device from the number of patients’ concerns. The details are presented in Table 5.

A negative emotionality correlated with all of the ICDC questionnaire subscales, and the higher the level of negative emotionality, the greater the intensity of anxiety for all of the ICDC subscales: number of concerns r = 0.58; *p* < 0.001, intensity of concern r = 0.61; *p* < 0.001, combined factor r = 0.61; *p* < 0.001, factor 1 r = 0.55; *p* < 0.001 and factor 2 r = 0.60; *p* < 0.001. There were similar observations for social inhibition. The greater the social inhibition, the higher the severity of the number of fears after ICD implantation: number of concerns r = 0.37; *p* < 0.001, intensity of concern r = 0.38; *p* < 0.001, combined factor: r = 0.39; *p* < 0.001, factor 1 r = 0.35; *p* < 0.001 and factor 2 r = 0.37; *p* < 0.001.

Disease acceptance, which was assessed after ICD implantation, correlated with all of the subscales of the ICDC questionnaire; the higher the level of disease acceptance, the smaller the number of fears and their severity: number of fears r = −0.58; *p* < 0.001, intensity of concern r = −0.63; *p* < 0.001, combined factor r = −0.62; *p* < 0.001, factor 1 r = −0.53; *p* < 0.001 and factor 2 r = −0.68; *p* < 0.001.

The symptoms of anxiety and depression contributed to the occurrence of anxiety in patients within six months after implantation. The greater the severity of anxiety, the higher the results of the ICDC questionnaire subscale (number of concerns r = 0.66, *p* < 0.001, intensity of concern r = 0.71, *p* < 0.001, combined factor r = 0.70, *p* < 0.001, factor 1 r = 0.63, *p* < 0.001 and factor 2 r = 0.70, *p* < 0.001). Similarly, the greater the severity of the depressive disorders, the greater the anxiety that was displayed by patients (number of fears r = 0.50, *p* < 0.001, severity of fears r = 0.54, *p* < 0.001, combined factor r = 0.53, *p* < 0.001, factor 1 r = 0.44, *p* < 0.001 and factor 2 r = 0.6, *p* < 0.001).

The occurrence of insomnia correlated with the severity and occurrence of fears after ICD implantation (number of fears r = 0.44, *p* < 0.001, severity of fears r = 0.46, *p* < 0.001, combined factor r = 0.46, *p* < 0.001, factor 1 r = 0.35, *p* < 0.001 and factor 2 r = 0.51, *p* < 0.001).

Additionally, a multiple regression model using the stepwise input method was performed. The predictors were age, symptoms of anxiety, symptoms of depression, acceptance of the disease and sleeplessness. The dependent variable was the number of concerns. The model was statistically significant and explained 42% of the observed variance in the dependent variable (*p* = 0.0001, R^2^ = 0.4215). The analysis showed that age (*p* = 0.0036), insomnia (*p* = 0.0276), anxiety (*p* = 0.0000) and negative emotions (*p* = 0.0374) were important predictors of the dependent variable and enabled more concerns to be predicted.

A multiple regression model in which the predictors were age, the symptoms of anxiety, depression, acceptance of the disease and insomnia and the dependent variable was the intensity of the concerns was statistically significant and explained 49% of the observed variance in the dependent variable (*p* = 0.0001; R^2^ = 0.4979). The analysis showed that age (*p* = 0.0008), insomnia (*p* = 0.0497) and anxiety symptoms (*p* = 0.0001) were important predictors of the dependent variable.

A multiple regression model in which the predictors were age, the symptoms of anxiety, depression, acceptance of the disease and insomnia and the dependent variable was the overall concerns score was statistically significant and explained 49% of the observed variance in the dependent variable (*p* = 0.0001; R^2^ = 0.4955). The analysis showed that age (*p* = 0.0005), anxiety symptoms (*p* = 0.0001) and insomnia (*p* = 0.0292) were important predictors of the dependent variable.

A multiple regression model in which the predictors were age, symptoms of anxiety, depression, acceptance of the disease and insomnia and the dependent variable was factor 1 was statistically significant and explained 39% of the observed variance in the dependent variable (*p* = 0.0001; R^2^ = 0.3941). The analysis showed that age (*p* = 0.0001) and anxiety symptoms (*p* = 0.0001) were important predictors of the dependent variable.

A multiple regression model in which the predictors were age, the symptoms of anxiety, depression, acceptance of the disease and insomnia and the dependent variable was factor 2 was statistically significant and explained 51% of the observed variance in the dependent variable (*p* = 0.0001; R^2^ = 0.5154). The analysis showed that the age (*p* = 0.0203), insomnia (*p* = 0.0021) and anxiety symptoms (0.0001) were important predictors of the dependent variable.

## 4. Discussion

The informational material for ICD patients largely focuses on problems that are related to the device itself, and the limitations on a patient’s lifestyle are not always discussed comprehensively. However, if these limitations are not addressed early after implantation, the avoidance of activities patients enjoy the most can lead to frustration in the long run. Avoiding activities and hobbies is also a risk for developing other diseases. The statements in the subscale questionnaire (factor 1 assesses the perceived limitations, and factor 2 assesses the device-specific concerns) provide better diagnostic options for the above-mentioned problems [10].

The ICDC questionnaire is a tool for the assessment of the number and severity of concerns in ICD patients. It can be used by healthcare professionals who are involved in the care of patients after implantation and, also, as a research tool. The study by Młynarska et al., a cross-cultural adaptation of ICDC, showed that the Polish language version of this questionnaire is valid and reproducible and can be used with Polish patients who have ICDs [11].

Along with the assessment of the concerns of patients with ICDs, other standardized questionnaires such as the DS 14, HADS, AIS, FCIS, ACDS and the Athens Insomnia Scale were also used.

In their study, Pedersen et al. examined the influence of type D personality and preimplantation device concerns on the short-term mortality in ICD patients. The median follow-up was two years. Patients with type D personality and high levels of preimplantation concerns had a poorer survival rate compared to patients with one or none of these risk marker [18]. In our study, patients with type D personality also had higher levels of concerns, but our study was performed six months after implantation, and we did not assess the mortality.

In another study by Pedersen et al., 32% of the patients experienced anxiety and 28% experienced depressive symptoms. Both symptoms were assessed using the HADS questionnaire and were more common for patients who expressed a high number of concerns in the ICDC [19]. Similarly, the present study found higher levels of anxiety and depressive symptoms among the patients who experienced a device intervention. The individuals who experienced a discharge had higher scores regarding the concerns in the ICDC questionnaire.

The aim of a cohort study by Versteeg et al. was to assess the frequency and markers of psychological distress in patients after ICD implantation. The incidence of anxiety was 16%, depression was 19% and 25% of patients reported one or both disorders in the first two weeks after implantation [20]. In our study, the evaluation was performed six months after implantation. Anxiety and depressive disorders were significantly more frequent in the group of patients that experienced defibrillator discharges.

Defibrillator cardioverter discharges are associated with concerns about an ICD and affect a number of patient behaviors, including treatment adherence, disease acceptance and chronic disease functioning. The questionnaire used to assess the concerns of an ICD patient is a new tool in the Polish language version.

In a study of the psychological status of 332 patients within 12 months after ICD implantation, Pedersen et al. showed that ICD shock and type D personality were independent predictors of deterioration in the psychological functioning in all of the domains. Patients with a primary prevention indication experienced a reduction in ICD anxiety and depression, and elderly patients had a reduction in anxiety during the follow-up period. In contrast, patients with left ventricular dysfunction were more likely to experience increased anxiety. In our study, according to Pedersen’s observations, elderly patients showed less intense concerns about ICD implantation. This age-related difference can be explained by the impact of less work duties and home matters on balance when considering the fears related to the return to their normal self. The reduced exercise fraction and the severity of heart failure symptoms, which were assessed on the NYHA scale, did not affect the severity of the concerns related to ICD implantation, which is a different result than the one that was obtained by Pedersen. However, patients who had indications for the implantation of a resynchronization device, which is for patients with more advanced heart failure, were excluded from our study. Our results were consistent with Pedersen’s results in terms of the impact of type D personality on the severity of anxiety and in the field of primary prevention, which caused a milder intensification of anxiety compared to the secondary prevention. Primary prophylaxis patients did not experience any negative effects of the disease, which resulted in no influence on the assessment of mental functioning [21].

In the present study, women had more intense concerns after ICD implantation. Our results are consistent with those presented by Vazquez et al. and Starrenburg et al., who presented results that indicated that women had a lower device acceptance than men because of concerns about body image. This is consistent with the qualitative descriptions of women who are embarrassed about wearing clothes that reveal their scars. More extensive education should be considered before the procedure of ICD implantation in women [22,23].

## 5. Conclusions

There is a relationship between the severity of the concerns related to an implanted ICD and age, anxiety, negative emotions and insomnia. A higher level of concern is also associated with female gender. Patients who experienced discharge had more concerns. The secondary prevention of sudden cardiac death may be associated with increased concerns about the ICD. A higher acceptance of illnesses resulted in a lower number of concerns and their severity. All patients should be more educated about the possible workings of the device (discharge), and psychosocial care after ICD implantation should be offered in some cases.

## Figures and Tables

**Table 1:** Characteristics of the study group.

Parameter	Value
Age (years)	67.61 ± 9.15
BMI (kg/m^2^)	29.01 ± 5.84
SBP (mmHg)	123.32 ± 12.87
DBP (mmHg)	74.8 ± 8.93
EF (%)	29.75 ± 8.34
HR (bpm)	72.84 ± 9.29
Marital status:	
Single	47 (29.7%)
Married/living with partner	111 (70.2%)
Education:	
None or primary	6 (3.8%)
Vocational	93 (58.9%)
Secondary	41 (25.9%)
Higher	18 (11.4%)
Disease:	
Heart Failure (NYHA I-IV)	140 (88.6%)
Hypertension	129 (81.6%)
Stroke	14 (8.8%)
Ischemic Heart Disease	123 (77.8%)
COPD	18 (11.3%)
Obesity	71 (44.9%)
Thyroid Disease	20 (12.6%)
Diabetes	67 (42.4%)
Medications:	
β-blocker	156 (98.1%)
ACE	144 (91.1%)
MRA	147 (93%)
Statins	136 (86%)
Oral antidiabetic drugs	63 (39.8%)
Insulin	26 (16.45%)

COPD—Chronic Obstructive Pulmonary Disease, ACE—Angiotensin-converting enzyme inhibitors and MRA—Mineralocorticoid receptor antagonists.

**Table 2:** Characteristics of the severity of the concerns about the implanted cardioverter defibrillator.

Number	Question I Am Worried About:	Not at All *N* (%)	A Little Bit *N* (%)	Somewhat *N* (%)	Quite a Lot *N* (%)	Very Much So *N* (%)
1.	My ICD firing	40 (25.31%)	53 (33.54%)	26 (16.45%)	22 (13.92%)	17 (10.75%)
2.	My ICD not working when I need it to	52 (32.91%)	59 (37.34%)	34 (21.51%)	8 (5.06%)	5 (3.16%)
3.	What I Should do if my ICD fires	67 (42.40%)	53 (33.54%)	29 (18.35%)	7 (4.43%)	2 (1.26%)
4.	Doing exercise in case it causes my ICD to fire	109 (68.98%)	27 (17.08%)	15 (9.49%)	5 (3.16%)	2 (1.26%)
5.	Doing activities/hobbies that may cause my ICD to fire	83 (52.53%)	35 (22.15%)	17 (10.75%)	11 (6.96%)	12 (7.59%)
6.	My heart condition getting worse if the ICD fires	106 (67.08%)	27 (17.08%)	11 (6.96%)	8 (5.06%)	6 (3.79%)
7.	The amount of time I spend thinking about my heart condition and having an ICD	144 (91.13%)	10 (6.32%)	4 (2.53%)	0 (0%)	0 (0%)
8.	The amount of time I spend thinking about my ICD firing	143 (90.5%)	10 (6.32%)	5 (3.16%)	0 (0%)	0 (0%)
9.	The ICD battery running out	108 (68.35%)	28 (17.72%)	14 (8.86%)	5 (3.16%)	3 (1.89%)
10.	Working too hard/overdoing things and causing my ICD to fire	76 (48.1%)	37 (23.41%)	22 (13.92%)	12 (7.59%)	11 (6.96%)
11.	Making love in case my ICD fires	104 (65.82%)	30 (18.98%)	13 (8.22%)	6 (3.79%)	5 (3.16%)
12.	Having no warning that my ICD will fire	32 (20.25%)	60 (37.97%)	20 (12.65%)	24 (15.18%)	22 (13.92%)
13.	The symptoms/pain associated with my ICD firing	59 (37.34%)	36 (22.78%)	20 (12.65%)	20 (12.65%)	23 (14.55%)
14.	Being a burden on my partner/family	106 (67.08%)	23 (14.55%)	17 (10.75%)	5 (3.16%)	7 (4.43%)
15.	Not being able to prevent my ICD from firing	126 (79.74%)	8 (5.06%)	11 (6.96%)	8 (5.06%)	5 (3.16%)
16.	The future now that I have an ICD	106 (67.08%)	16 (10.12%)	14 (8.86%)	14 (8.86%)	8 (5.06%)
17.	Problems occurring with my ICD, e.g., battery failure	86 (54.43%)	29 (18.35%)	22 (13.92%)	12 (7.59%)	9 (5.69%)
18.	Getting too stressed in case my ICD fires	141 (89.24%)	7 (4.43%)	3 (1.89%)	4 (2.53%)	3 (1.89%)
19.	Not being able to work/take part in activities and hobbies because I have an ICD	131(82.91%)	13 (8.22%)	4 (2.53%)	6 (3.79%)	4 (2.53%)
20.	Exercising too hard and causing my ICD to fire	144 (91.13%)	7 (4.43%)	3 (1.89%)	2 (1.26%)	2 (1.26%)

ICD—Implantable cardioverter defibrillator.

**Table 3:** Correlations between age and concerns on the ICDC scale.

Parameter	Age Spearman Correlation Coefficient	
Number of concerns	r = −0.282	*p* < 0.001
Intensity of concerns	r = −0.278	*p* < 0.001
Overall concerns score	r = −0.28	*p* < 0.001
Factor 1-perceived limitations	r = −0.357	*p* < 0.001
Factor 2-device specific concerns	r = −0.209	*p* = 0.008

*p*—Mann–Whitney test.

**Table 4:** Gender and concerns after ICDC implantation.

Parameter	Gender—Value		
	Women (*N* = 31)	Men (*N* = 127)	*p*
Number of concerns	9.84 ± 5.09	7.02 ± 4.8	*p* = 0.006
Intensity of concerns	18.81 ± 13.52	13.46 ± 14.19	*p* = 0.012
Overall concerns score	28.65 ± 17.93	20.48 ± 18.63	*p* = 0.009
Factor 1—perceived limitations	5.32 ± 5.17	4.9 ± 6.4	*p* = 0.162
Factor 2—device specific concerns	10.48 ± 7.44	7.14 ± 7.75	*p* = 0.005

*p*—Mann–Whitney test.

**Table 5:** The type of prevention versus the number and intensity of any fears.

Parameter	Prevention—Value		
	Primary	Secondary	*p*
Number of concerns	7.24 ± 4.85	9.48 ± 5.32	*p* = 0.06
Intensity of concerns	13.21 ± 13.08	22.13 ± 17.96	*p* = 0.022 *
Overall concerns score	20.46 ± 17.48	31.61 ± 23.05	*p* = 0.028 *
Factor 1—perceived limitations	4.46 ± 5.66	8.04 ± 8.03	*p* = 0.016 *
Factor 2—device specific concerns	7.05 ± 7.12	12.17 ± 10	*p* = 0.026 *

*p*—Mann–Whitney test. * Statistically significant relationship (*p* < 0.05).

## Data Availability

Data sharing not applicable.

## Study Limitation

This study was a single-center study, and the patients included in the study were from one region. Moreover, only a relatively small group of patients was included in the study. The period of observation of patients after ICD implantation could be extended.
